# Antenatal Depressive Symptoms and Neurodevelopment Outcomes in Children at 30 Months. A Study From South India

**DOI:** 10.3389/fpsyt.2020.486175

**Published:** 2020-09-24

**Authors:** Susan Thomas, Emelia Vigil, Tinku Thomas, David C. Bellinger, Asha Ramthal, Anura V. Kurpad, Christopher P. Duggan, Krishnamachari Srinivasan

**Affiliations:** ^1^Division of Mental Health & Neurosciences, St John’s Research Institute, Bengaluru, India; ^2^Department of Psychology, Harvard University, Cambridge, MA, United States; ^3^Division of Epidemiology, Biostatistics and Population Health, St John’s Research Institute, Bengaluru, India; ^4^Department of Neurology, Boston Children’s Hospital, Harvard Medical School, Boston, MA, United States; ^5^Division of Nutrition, St John’s Research Institute, Bengaluru, India; ^6^Department of Physiology, St Johns Medical College, Bengaluru, India; ^7^Center for Nutrition, Division of Gastroenterology, Hepatology and Nutrition, Boston Children’s Hospital, Boston, MA, United States; ^8^Department of Psychiatry, St John’s Medical College, Bengaluru, India

**Keywords:** antenatal depressive symptoms, neurodevelopment, language, education, social support, coping

## Abstract

**Background:**

Prevalence of antenatal depression in low and middle income countries is high. However studies examining the association between maternal antenatal depression and early childhood development from these countries are scarce. The objective of the study was to examine the association between antenatal depressive symptoms assessed serially during pregnancy and child neurodevelopment outcomes in mother–child dyads part of a randomized control trial of maternal B12 supplementation during pregnancy.

**Method:**

Subjects were 203 women who had participated in the placebo-controlled, randomized trial of vitamin B12 supplementation during pregnancy and 6 weeks post-partum on whom serial assessments of depressive symptoms in each of the trimesters were available. Cognitive, receptive language, expressive language, fine motor skills and gross motor skills were assessed at 30 months using the Bayley’s Scale of Infant Development-3rd edition (BSID-III). Antenatal depressive symptoms were assessed at three trimesters using the Kessler’s 10 Psychological Distress Scale (K10). Women were classified into three categories: not depressed (K10 <6 in all trimesters), with intermittent depressive symptoms (K10 ≥6 in at least one trimester) and with persistent depressive symptoms (K10 score ≥6 in at least 2 trimesters).

**Results:**

112 (55.2%) of the women did not have depressive symptoms, 58 (28.6%) had intermittent depressive symptoms and 33 (16.2%) had persistent depressive symptoms. The children of women with intermittent antenatal depressive symptoms scored lower on the receptive language domain on BSID-III compared to children of women who were not depressed on univariate analysis, but not on bivariate regression analysis. Women with persistent depressive symptoms had lower educational attainment (p = 0.004), lower social support (p = 0.006) and used more emotional coping strategies (p = 0.005) compared to the not depressed group.

**Conclusions:**

A significant number of women in south India had antenatal depressive symptoms. Findings from this study suggest a possible association between antenatal depressive symptoms and receptive language in children. Larger studies including women with clinical depression are needed to confirm these findings.

## Introduction

Higher maternal stress and anxiety during pregnancy are associated with poorer cognitive development in infants (1) and young children (2). Children of women with persistent depression during pregnancy had higher odds of developmental delays at 18 months on the Denver Developmental Screening Test (adjusted odds ratio 1.34, 95% CI 1.11, 1.62) (3). In a prospective study of 1030 mother-child pairs from the US, children of women with depression at mid-pregnancy had lower scores on Peabody Picture Vocabulary Test (PPVT) at 30 months (4). In contrast, in a study among US Caucasian women, a mild to modest level of a composite score of maternal depression measured at 24 weeks of pregnancy was associated with higher cognitive scores on Bayley’s Scale of Infant Development (BSID) II in children (n = 82) at 24 months (5). A recent study concluded that antenatal anxiety but not depression was associated with poorer cognitive outcomes in 3 year old children (6).

The prevalence of antenatal depressive symptoms is high among women in the Asian subcontinent (7, 8). However, studies examining the association between maternal antenatal depression and early childhood development from resource-poor settings are scarce. In a study from Ethiopia, depressive, anxiety and somatic symptoms during pregnancy were associated with poorer infant motor development on BSID-III in infants at 12 months but this association became non-significant after adjusting for socioeconomic, maternal and pregnancy related factors (9).

A recent review highlighted the mutual influence of maternal antenatal depression and nutritional status and their effect on child neurocognitive development (10). The authors recommend including both exposures in study designs to determine their relative and/or additive impact on child neuro development. The effects of maternal Iron Deficiency Anemia (IDA) and Common Mental Disorders (CMD) during pregnancy on child development found that a combined effect of maternal IDA and CMD resulted in a larger reduction in the cognitive domain on BSID III, than the individual effects (11). Vitamin B12 and its metabolites have also been linked to maternal depression and child neurodevelopment (12). The GUSTO study (13) examined the relationship of plasma folate and B12 levels to antenatal depression in 709 women in Singapore. They observed that plasma folate, but not B12 levels, was significantly lower in those with probable depression. Neurodevelopmental outcomes were measured on 443 children from this cohort at 2 years using BSID III (14) and compared across groups based on maternal B12 status, deficient(<148 pmol/L), insufficient (148–220.9 pmol/L) and sufficient (>221 pmol/L). Infants of mothers with vitamin B12 deficiency had 0.42 SD lower scores on the cognitive domain of BSID III when compared to infants of mothers with sufficient levels of B12. There was no additive effect of antenatal depression on cognitive outcomes.

The effects of maternal antenatal depression on child cognitive or temperamental outcomes could be trimester specific due to programming effects depending on the developmental age and sensitive periods (15). In addition, chronic maternal depression has been linked to poorer neurodevelopmental outcomes (3, 16). Hence it is important to assess maternal depression at multiple time points during pregnancy.

Results from previous studies that examined the effects of depressive symptoms during pregnancy on child cognitive outcomes are inconsistent. The majority of these studies have measured depression at only one time point during pregnancy. A recent study that investigated prenatal/post natal trajectories of maternal anxiety measured at multiple time points during pregnancy and in the post natal period found that only high persistent anxiety was predictive of developmental delay in children at 3 years (17). It is also seen in previous studies that maternal nutrition, especially vitamin B12, its metabolites and folate could have an association with maternal depression and child neurodevelopment. Thus examining the impact of maternal antenatal depressive symptoms and nutritional status on child cognitive outcomes is particularly relevant to low and middle income countries (LMIC) as the rates of antenatal depression and inadequate nutritional status during pregnancy are high. We performed a double-blind, placebo-controlled trial of oral vitamin B12 during pregnancy and early lactation in South Indian women (18). As part of this trial, antenatal depressive symptoms were measured at three trimesters in pregnancy. The objective of the present study was to examine the association between antenatal depressive symptoms, assessed serially during pregnancy and neurodevelopment outcomes in children. We hypothesized that children born to mothers with persistent depression will perform poorer on BSID-III compared to mothers who did not report any depressive symptoms during pregnancy. In addition, we also compared the plasma levels of B12 and its metabolites during pregnancy in women with and without depressive symptoms.

## Materials and Methods

**Figure 1:**
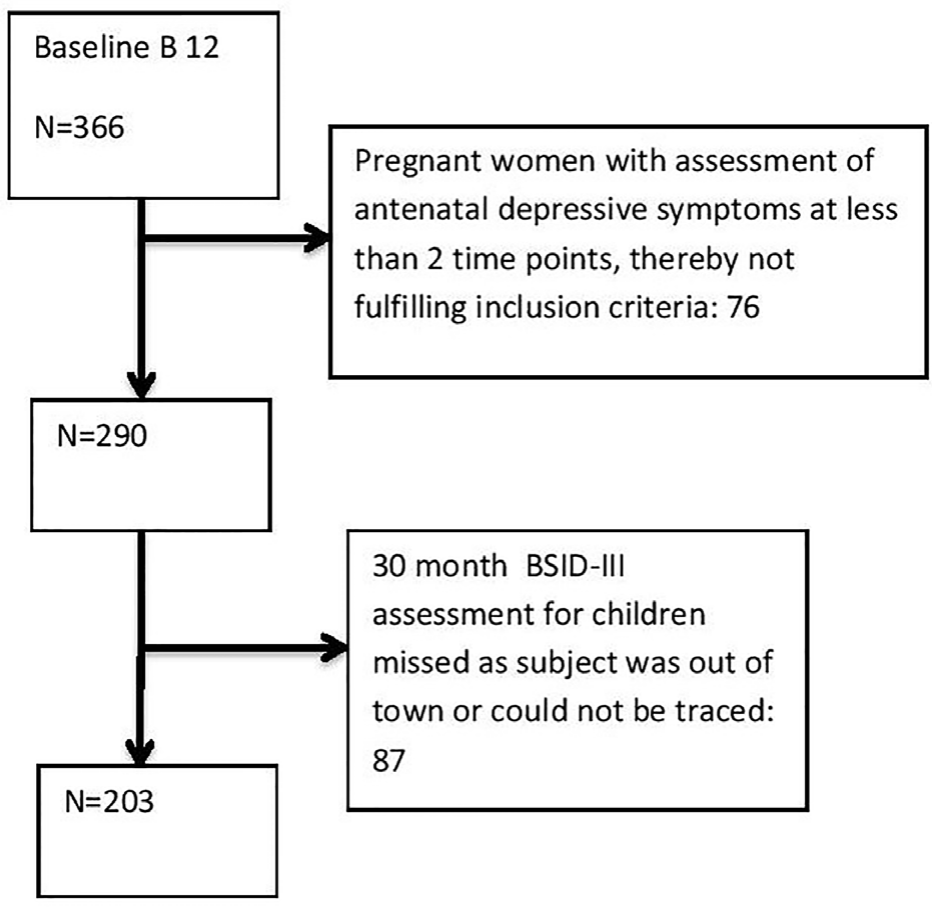
Flowchart of sample inclusion from baseline to assessment of children at 30 months.

Women and children who enrolled in the parent placebo-controlled, randomized trial of vitamin B12 supplementation during pregnancy and 6 weeks post-partum were eligible for inclusion in this study. The parent randomized controlled trial was registered at clinicaltrials.gov as NCT00641862. The present study was approved by the Institutional Ethics Committee, St. John’s National Academy of Health Sciences, Bangalore and the Institutional Review Board of the Harvard T. H. Chan School of Public Health. The recruitment site was Hosahalli Hospital, a government hospital serving an undeserved community from urban Bangalore. Eligibility for the parent trial included women aged ≥18 years who had registered for antenatal care at/before 14 weeks gestational age. Excluded were women with multiple gestations, chronic medical conditions, those who anticipated moving out of the area before study completion, those who tested positive for hepatitis B (HepBsAg), HIV or syphilis (VDRL) infections, and those who were already taking daily vitamin supplements in addition to folate and iron. The women were scheduled to be screened by Kessler psychological distress scale K-10) during the three trimesters in the parent trial. Women with severe depressive symptoms (K-10 scores ≥ 20) were referred to a government psychiatric facility for further evaluation and treatment. The present study used a convenient sample from the parent trial that consisted of women who were assessed for depressive symptoms at a minimum of 2 out of 3 time points during the antenatal period (**Figure 1**).

The women were approached for the 30 month cognitive assessment of their children and consent was sought. 203 mother-child pairs participated in the study. After written informed consent was obtained for the assessment, a study research assistant reviewed the baseline socioeconomic and demographic data that was collected upon initial entry into the study (**Table 1**).

**Table 1:** Socio-demographic characteristics, birth outcomes and K10 scores of pregnant women whose children underwent BSID-III testing at 30 months of age.

Parameters	Not Depressed (n = 112)	Intermittently Depressed (n = 58)	Persistently Depressed (n = 30)	p value
Age, (years)	27.4 ± 3.8^1^	26.4 ± 3.6^1^	26.8 ± 3.3^1^	0.18
Level of education				0.004
Up to middle school	16 (15.8)^2^	15 (28.3)^2^	14 (46.7)^2^	
High school	50 (49.5)^2^	28 (52.8)^2^	11 (36.7)^2^	
Post high school	35 (34.7)^2^	10 (18.9)^2^	5 (16.7)^2^	
Monthly household income, INR	7000 (5000,10000)^3^	8000 (5000,10000)^3^	5500 (4000,9250)^3^	0.30
Maternal employment				0.99
Employed	13 (13)^2^	7 (13)^2^	4 (13)^2^	
Unemployed	88 (87)^2^	46 (87)^2^	26 (87)^2^	
Parity				0.64
0	56 (54.4)^2^	39 (73.6)^2^	19 (63.3)^2^	
≥ 1	47 (45.6)^2^	14 (26.4)^2^	11 (36.7)^2^	
Sex of the baby				0.76
Male	45 (46)^2^	25 (51)^2^	12 (43)^2^	
Caesarian section	32(28.6)^3^	12(20.7)^3^	8(26.7)^3^	0.51
Gestational age at birth	39.2(38.4,39.6)^2^	39.2(38.1,39.6)^2^	39.0(38.2, 40.0)^2^	0.83
Infant birthweight (kg)	3.0 (2.5,3.25)^3^	2.85 (2.53, 3.23) ^3^	2.75 (2.5,3) ^3^	0.36
IUGR	31(27.7)^2^	19(32.8)	12(40)	0.65
Mean (SD) K 10 score	1 (0.1)^3^	4 (3,5)^3^	9 (6,12)^3^	<0.001

^1^Mean ± SD.

^2^N(%).

^3^Median (Q1, Q3).

IUGR, intrauterine growth retardation (birth weight less than the 10th percentile of norms for gestational age).

### Assessment of Child Neurodevelopment

The Bayley Scales of Infant Development, 3rd edition, (BSID-III) was used to assess infant neurodevelopment status at 30 months of age ± 2 weeks. The scales assess five domains: cognitive, language (receptive and expressive), motor (fine and gross), social-emotional, and adaptive (conceptual, social, practical). The latter two domains were not assessed as they were deemed not culturally appropriate. BSID III is a widely used instrument for the measurement of neurocognitive assessment in children at this age (5, 9, 11, 14). The instructions for the test items were translated into the local language (Kannada). Two master’s level psychologists experienced in child developmental testing, trained in the administration and scoring of BSID-III by an international expert (DB) and blind to group assignment administered the tests. We used raw scores from BSID-III for comparing the groups since age-specific norms are not available for Indian children. The BSID-III raw scores were adjusted for the children’s gestational age at birth.

### Assessment of Psychological Factors

#### Kessler Psychological Distress Scale

Depressive symptoms were assessed using Kessler Psychological Distress Scale (K-10). The K-10 consists of 10 items based on a 4-week recall period with each item having five response categories and is scored from 0 to 4 (19). In the present study, we administered a translated version of K-10 in local language (Kannada) used in previous studies of assessment of antenatal depressive symptoms (7, 20). In a previous study among South Indian pregnant women, K-10 was found to be good screening instrument for identifying antenatal depression in South India at a cutoff score ≥6 (sensitivity = 100%, specificity = 81.3%, and area under the curve = 0.95). A high degree of correlation was also found between Edinburgh Postnatal Depression Scale (EPDS) and K10 scores (Spearman’s r = 0.67; p<0.01) (20). It was noted in the study that participants had difficulty in comprehending some of the items on EPDS and the use of different response options per item question on the EPDS as compared with the same Likert structure for all item options on the K10. For these reasons, we chose to use K10 as the demographic characteristics of the participants in the present study were similar to the population in the original study that compared K10 and EPDS. Women who had K-10 scores ≥ 6 in 2 or more trimesters of pregnancy were categorized as persistently depressed. Women with K10 scores <6 at all trimesters belonged to the non-depressed category, and women with K10 scores ≥ 6 in any one trimester were defined as intermittently depressed.

#### Social Support Scale

A questionnaire in local language was used to measure a broad range of social support available to the woman (21). The questionnaire consisted of 12 items; six reflecting instrumental and six reflecting emotional support with options ranging from definitely not enough (score = 1) to definitely enough (score = 4). This scale was administered in the second trimester.

#### Coping Checklist

This 70 item scale scored dichotomously as “yes” or “no” indicating the presence or absence of a particular coping behavior (22). The test retest reliability was 0.74. The scale gives information on whether the person uses problem solving coping or emotional coping strategies and has been used in the Indian setting in a variety of populations including women (23). This scale was administered in the second trimester.

### Assessment of Dietary Intake During Pregnancy

A pretested interviewer-administered Food Frequency Questionnaire (FFQ) (24) was used to assess habitual dietary intake during the three trimesters. Standard measures were placed before the respondent to quantify the portion size of each food item when administering the FFQ by trained interviewers. Nutrient information was obtained for 27 macronutrients and micronutrients. We compared energy, carbohydrate, protein, and fat intakes and micronutrients riboflavin, vitamin B6, vitamin B12, folate, and iron intake averaged over three trimesters across the three groups of women.

### Assessment of Biochemical Parameters During Pregnancy

Details of biochemical assays have been previously described (18). Briefly, 10 ml of blood was obtained from the women by venipuncture at 12 weeks (baseline), 24 weeks and 33 weeks of pregnancy. The plasma and RBCs were separated and stored at −80°C until analysis. Plasma vitamin B12 was measured by electrochemiluminescence (Roche Diagnostics Mannheim, USA). The intraday and interday assay CVs for vitamin B12 were 0.54% and 2.44% respectively. Plasma total homocysteine (tHcy) and methyl malonic acid (MMA) were estimated by GC-MS (model 3800; Varian, Palo Alto, CA, USA). The interday assay CVs for tHcy and MMA were 5.04% and 5.57% respectively and the intraday assay CVs were 5.60% and 6.92%, respectively. Erythrocyte folate was measured by a competitive immunoassay with direct chemiluminescence detection on an automated immunonanalyzer (ADVIA Centaurs, Tarrytown, New York, USA). The intra-assay and inter-assay variabilities were 1.9% and 5.2%, respectively. The following cutoff values were used: low vitamin B12 as <150 (pmol/L), elevated MMA as > 0.26 (µmol/L) and elevated tHcy as >15.0 (µmol/L).

## Statistical Analysis

We used SPSS Version 22 for all analyses. Continuous data were described using mean ± SD and categorical data using n (%). The normality of the data was examined by graphically evaluating Q–Q plots. Kruskal Wallis test was used to compare the three groups of women on biochemical parameters, psychological and cognitive measures as they were skewed. Post-hoc comparisons were done using Mann-Whitney U-test with Bonferroni adjusted p values. Bivariate linear regression analysis was performed if the initial non-parametric comparisons were statistically significant. Regression coefficients (β) and corresponding 95% confidence interval (95% CI) are reported. The level of signiﬁcance used for statistical significance was P<0.05.

## Results

Of the 365 women who were recruited in the parent trial, 203 mother-child dyads were eligible for the present study (**Figure 1**). There were no differences in the socio demographic characteristics or baseline biochemical characteristics of the women included in the study and those who were excluded (data not shown). One hundred and twelve women (55.2%) did not report depressive symptoms in any of the trimesters (not depressed group), while 58 women (28.6%) reported depressive symptoms at one trimester (intermittent depressed group) and thirty three women (16.2%) had depressive symptoms in at least 2 trimesters (persistent depressed group).

Women with persistent depressive symptoms had a lower educational attainment compared to the other two groups of women (**Table 1**). Maternal age, family income, parity, maternal employment, sex of the infant and birth outcomes that included mode of delivery, birth weight, gestational age at birth and intrauterine growth restriction were not associated with the presence of persistent depressive symptoms during pregnancy (**Table 1**). Dietary intakes were not significantly different between the three groups of women (data not presented).

Nutritional markers during pregnancy (vitamin B12, MMA, tHcy, erythrocyte folic acid and Hb) across the three trimesters were not significantly different among the three groups of women (**Table 2**).

**Table 2:** Biochemical characteristics of pregnant women whose children underwent BSID-III testing at 30 months of age.

Variables	Not Depressed (n = 112)	Intermittently Depressed (n = 58)	Persistently Depressed (n = 30)	p value
Vitamin B12 pmol/L	149.7(104.3,204.1)	166.3 (118.1,261.7)	148.1 (112.4,188.9)	0.16
MMA 0.26 μmol/L	0.4 (0.3,0.6)	0.4 (0.2,0.7)	0.4 (0.2,0.6)	0.94
tHcy 15.0 μmol/L	7.6 (4.4,11.7)	7.6 (5.0,11.5)	8.21 (5.5, 11.8)	0.67
Erythrocyte folic acid, nmol/L	457.9 (385.2, 552.58)	494.3 (401.7, 609.4)	507.6 (409.6, 605.7)	0.40
Hemoglobin g/dl	11.0 (10.0,11.7)	11.2 (10.5, 11.9)	11.0 (10.0,11.7)	0.24

Median (Q1, Q3) values across trimesters.

MMA, methylmalonic acid.

The number of women who had low scores on social support scale was significantly higher in the persistently depressed group. Women with persistent depressive symptoms used more emotional coping mechanisms compared to women in the not depressed group (**Table 3**).

**Table 3:** Psychological characteristics of the three groups of pregnant women whose children underwent BSID-III testing at 30 months of age.

Variables	Not Depressed (n = 112)	Intermittently Depressed (n = 58)	Persistently Depressed (n = 30)	p value
Social Support	(Social support scale)			
Instrumental	1 (0.9)^1^	5(8.6)^1^	4(13.3)^1^	0.02
Emotional	2 (1.8)^1^	3 (5.2)^1^	9 (30)^1^	<0.001
Total	2 (1.8)^1^	6 (10.3)^1^	7(23.3)^1^	0.01
Coping mechanisms	(Coping Checklist)			
Problem solving	5 (4,7)^2^	6 (4,7)^2^	5 (4,7)^2^	0.58*
Emotional	21 (18,24)^2^	23 (20,25)^2^	23 (21,26)^2^	0.01*
Seeking social support	5 (3,5)^2^	5 (4,5)^2^	5 (4,5)^2^	0.91*
Total	31 (28,34)^2^	33 (29,36)^2^	33 (30,36)^2^	0.03*

^1^N(%) of subjects who scored below median.

^2^Median (Q1, Q3) values across trimesters.

p value from Chi square test.

*p value from Kruskal Wallis test.

The receptive language sub-domain score was significantly lower in children of women with intermittent depressive symptoms compared to children of women with no depressive symptoms and was similar to the score of children of women with persistent depressive symptoms (**Table 4**). However, the difference in receptive language scores on BSID-III was not significantly different between the three groups of women in a bivariate linear regression analysis (*B* = 1.42, 95% CI: (−0.43, 3.28 for “no depression” vs “intermittent depression” and *B* = 1.87, 95% CI: −0.61, 4.34 for “persistent depression” vs “intermittent depression”) after adjusting for total scores for coping and social support.

**Table 4:** BSID III subdomain scores at 30 months of the children of the three groups of pregnant women based on depressive symptoms.

Domain	Not Depressed (n = 112)	Intermittently Depressed (n = 58)Median(Q1, Q3)	Persistently Depressed (n = 33)	p value
Cognitive	72 (69, 75)	71.5 (69.75, 73)	71 (69, 75)	0.62
Receptive language	38 (34, 41)	35.5 (32, 39)	37 (34, 40)	0.04
Expressive language	37 (34, 38)	35 (32, 38)	36 (33, 38)	0.10
Fine motor skills	47 (45, 49)	46 (44, 48)	47 (44, 49)	0.22
Gross motor skills	68 (64, 69)	67 (62, 69)	66 (63, 68)	0.29

p value from Kruskal Wallis test.

## Discussion

We found that a significant proportion of pregnant women in South India had depressive symptoms across multiple time points of assessment during pregnancy. Antenatal maternal depressive symptoms were associated with poorer child performance on receptive language domain on BSID-III on univariate analysis, though this association did not emerge on a bivariate regression analysis. Women with persistent depressive symptoms had lower educational attainment and reported lower social support (instrumental and emotional) and used more emotional coping strategies compared to women who were not depressed or who were intermittently depressed. There were no significant differences in average dietary intakes across the three trimesters of pregnancy among the three groups of women. There were no significant differences in plasma B12, elevated tHcy, MMA, erythrocyte folate levels and anemia among the three groups of women.

A high prevalence rate of antenatal depressive symptoms in our study is consistent with earlier observations among pregnant women from low and middle income countries (LMIC) (7, 8). Our finding that women with persistent depressive symptoms were less educated than women in the not depressed group is in agreement with earlier studies that noted that depressive symptoms are greater in pregnant women with low educational achievements (25, 26). Less social support in general and less emotional support in particular was associated with persistence of depressive symptoms as noted in previous studies (27, 28). In a community based study from Pakistan, it was observed that availability of social support from extended family network was associated with a decreased risk for depression during pregnancy and in the peri-natal period (29). This ability to draw support from family members is also linked to level of maternal education as women with higher educational attainment seemed to be better equipped to garner support from key family members (30).

Our findings of a possible association between maternal antenatal depressive symptoms and poorer receptive language domain scores on BSID-III in children at 30 months are in agreement with several earlier studies. Studies done in the US, Ethiopia and Ireland also noted an association between antenatal depression and poorer cognitive performance of children, but these effects were largely attenuated when adjusted for various socio demographic characteristics (4, 9, 31). In the present study, we did not do a longitudinal assessment of maternal post natal depressive symptoms which could have impacted child cognitive functions. Several studies have noted that postnatal maternal depression could have an adverse influence on child cognitive outcomes due to poorer mother child interaction and less cognitive stimulation at home (32–34). A longitudinal study with a larger sample size that measures both antenatal and post natal depressive symptoms may be able to reliably assess the cause and effects of maternal depression on cognitive performance of children.

In the present study, the effect of maternal antenatal depressive symptoms on receptive language domain was seen only among children of women who had intermittent depressive symptoms, compared to children of mothers who had no depressive symptoms or persistent depressive symptoms. This is in contrast to an earlier study that noted a higher risk of development delay in children born to women who had depressive symptoms at two time points in pregnancy compared to children of mothers with depression at any one time point during pregnancy (3). However, in this study the cognitive measures were based on the mothers’ self-report and maternal mood could have biased parental report on cognitive outcomes in children. The association between poorer performance on receptive language domain and intermittent maternal antenatal depressive symptoms as opposed to persistent maternal antenatal depressive symptoms suggests a possibility of trimester specific effect of maternal antenatal depressive symptoms on child cognitive outcomes (15). While fetal brain development occurs throughout pregnancy, recent neuroimaging studies have shown that there is a spurt in fetal brain development in the third trimester of pregnancy that includes the beginning of myelination, neuronal organization, spinogenesis and synaptogenesis and increase in brain volume (35, 36). In a recent study, we observed that some maternal nutrient markers in the third trimester as compared to the first and second trimesters were associated with amplitude of P300, a neurophysiological marker of cognitive function in children at 72 months (37). However, in the present study, our sample was limited to allow for examining trimester specific associations between antenatal depressive symptoms and child cognitive outcomes.

We did not find any difference in vitamin B12 levels, elevated tHcy, and MMA, and erythorocyte folate levels and anemia among the three groups of women. We also did not find any significant differences in dietary intake among the three groups of women. Recent systematic reviews of the associations between maternal nutritional biomarkers and depression and/or anxiety during pregnancy found that there was a high variability in the associations and opined that further studies in nutrient deficient populations were needed to draw firm conclusions (38, 39).

The findings from the present study of an association between maternal antenatal depressive symptoms and poorer performance in the receptive language domain should be interpreted with caution as BSID III was not adapted for use in the Indian setting and age specific norms are not available. A recent study (40) from Nepal found that the distribution of scores on BSID III were similar to the US norms, except for the language domain. However, in the present study we did not intend to assess individual children for developmental delays and earlier studies have used BSID III raw scores to examine the impact of antenatal factors on child cognitive performance (14, 41).

The major strength of our study is that we assessed depressive symptoms at each of the three trimesters of pregnancy. The neurodevelopment assessments were carried out by trained child psychologists with considerable experience in the administration of BSID-III. We also captured several psychosocial variables linked to maternal antenatal depressive symptoms such as social support and coping mechanisms. The use of a screening measure to assess maternal depression as opposed to a clinical assessment of depression is an important limitation. We could not access the details of the treatment offered to women who were referred to a government facility for treatment of severe depressive symptoms.

## Conclusions

A significant proportion of pregnant South Indian women had antenatal depressive symptoms. We found a modest association between maternal depressive symptoms and impairment in receptive language domain in children on a univariate analysis, but could not be confirmed on bivariate regression analysis. The possible negative association between antenatal depressive symptoms and receptive language domain assumes importance given the sub-optimal cognitive outcomes in children from LMIC. With high prevalence of prenatal depression in low and middle income countries and the relatively large population of children in these countries, the effects of prenatal depressive symptoms on child cognitive outcomes could be magnified, thus contributing to a significant burden to the society. Future longitudinal studies with a larger sample size will be helpful to examine this association between antenatal depressive symptoms and child cognitive outcomes.

## Data Availability Statement

The datasets generated for this study are available on request to the corresponding author.

## Ethics Statement

The studies involving human participants were reviewed and approved by Institutional Ethics Committee, St. John’s National Academy of Health Sciences, Bangalore and the Institutional Review Board of the Harvard T. H. Chan School of Public Health. Written informed consent to participate in this study was provided by the participants’ legal guardian/next of kin.

## Author Contributions

KS, AK, and CD conceptualized the project. AR conducted the psychological assessments and collected data. KS and ST were responsible for setting up the study. DB and ST supervised cognitive and psychological assessments. TT designed the statistical analyses. ST, EV, and KS wrote the paper. KS had the primary responsibility for the final content. All authors contributed to the article and approved the submitted version.

## Funding

This work was supported by Indian Council of Medical Research grant [5/7/900/12-RCH]. The parent trial was supported by the Indian Council of Medical Research grant [5/7/192/06-RHN]; and the US National Institutes of Health grant [R03 HD054123]. CD was supported in part by NIH grants [K24 DK104676, P30 DK040561].

## Conflict of Interest

The authors declare that the research was conducted in the absence of any commercial or financial relationships that could be construed as a potential conflict of interest.

## References

[1] Buitelaar JK Huizink AC Mulder EJ de Medina PGR Visser GHA Prenatal stress and cognitive development and temperament in infants. Neurobiol Aging (2003) 24 Suppl 1:S53–60; discussion S67-68. 10.1016/S0197-4580(03)00050-2 12829109

[2] Brouwers EPM van Baar AL Pop VJM Maternal anxiety during pregnancy and subsequent infant development. Infant Behav Dev (2001) 24:95–106. 10.1016/S0163-6383(01)00062-5

[3] Deave T Heron J Evans J Emond A The impact of maternal depression in pregnancy on early child development. BJOG Int J Obstet Gynaecol (2008) 115:1043–51. 10.1111/j.1471-0528.2008.01752.x 18651886

[4] Tse AC Rich-Edwards JW Rifas-Shiman SL Gillman MW Oken E Association of maternal prenatal depressive symptoms with child cognition at age 3 years. Paediatr Perinat Epidemiol (2010) 24:232–40. 10.1111/j.1365-3016.2010.01113.x PMC2860615 20415752

[5] DiPietro JA Novak MFSX Costigan KA Atella LD Reusing SP Maternal psychological distress during pregnancy in relation to child development at age two. Child Dev (2006) 77:573–87. 10.1111/j.1467-8624.2006.00891.x 16686789

[6] Ibanez G Bernard JY Rondet C Peyre H Forhan A Kaminski M Effects of antenatal maternal depression and anxiety on children’s early cognitive development: A Prospective Cohort Study. PloS One (2015) 10:e0135849. 10.1371/journal.pone.0135849 26317609 PMC4552796

[7] Lukose A Ramthal A Thomas T Bosch R Kurpad AV Duggan C Nutritional factors associated with antenatal depressive symptoms in the early stage of pregnancy among urban South Indian women. Matern Child Health J (2014) 18:161–70. 10.1007/s10995-013-1249-2 23440491

[8] Satyanarayana VA Lukose A Srinivasan K Maternal mental health in pregnancy and child behavior. Indian J Psychiatry (2011) 53:351–61. 10.4103/0019-5545.91911 PMC3267349 22303046

[9] Servili C Medhin G Hanlon C Tomlinson M Worku B Baheretibeb Y Maternal common mental disorders and infant development in Ethiopia: the P-MaMiE Birth Cohort. BMC Public Health (2010) 10:693. 10.1186/1471-2458-10-693 21073710 PMC3091583

[10] Monk C Georgieff MK Osterholm EA Research review: maternal prenatal distress and poor nutrition - mutually influencing risk factors affecting infant neurocognitive development. J Child Psychol Psychiatry (2013) 54:115–30. 10.1111/jcpp.12000 PMC3547137 23039359

[11] Tran TD Biggs B-A Tran T Simpson JA Hanieh S Dwyer T Impact on infants’ cognitive development of antenatal exposure to iron deficiency disorder and common mental disorders. PloS One (2013) 8:e74876. 10.1371/journal.pone.0074876 24086390 PMC3781140

[12] Dominguez-Salas P Cox SE Prentice AM Hennig BJ Moore SE Maternal nutritional status, C(1) metabolism and offspring DNA methylation: a review of current evidence in human subjects. Proc Nutr Soc (2012) 71:154–65. 10.1017/S0029665111003338 PMC3491641 22124338

[13] Chong MFF Wong JXY Colega M Chen L-W van Dam RM Tan CS Relationships of maternal folate and vitamin B12 status during pregnancy with perinatal depression: The GUSTO study. J Psychiatr Res (2014) 55:110–6. 10.1016/j.jpsychires.2014.04.006 24774647

[14] Lai JS Mohamad Ayob MN Cai S Quah PL Gluckman PD Shek LP Maternal plasma vitamin B12 concentrations during pregnancy and infant cognitive outcomes at 2 years of age. Br J Nutr (2019) 121(11):1303–12. 10.1017/S0007114519000746 PMC6660314 30935438

[15] Davis EP Sandman CA The timing of prenatal exposure to maternal cortisol and psychosocial stress is associated with human infant cognitive development. Child Dev (2010) 81:131–48. 10.1111/j.1467-8624.2009.01385.x PMC2846100 20331658

[16] Brennan PA Hammen C Andersen MJ Bor W Najman JM Williams GM Chronicity, severity, and timing of maternal depressive symptoms: relationships with child outcomes at age 5. Dev Psychol (2000) 36:759–66. 10.1037/0012-1649.36.6.759 11081699

[17] Mughal MK Giallo R Arnold P Benzies K Kehler H Bright K Trajectories of maternal stress and anxiety from pregnancy to three years and child development at 3 years of age: findings from the All Our Families (AOF) pregnancy cohort. J Affect Disord (2018) 234:318–26. 10.1016/j.jad.2018.02.095 29604550

[18] Duggan C Srinivasan K Thomas T Samuel T Rajendran R Muthayya S Vitamin B-12 supplementation during pregnancy and early lactation increases maternal, breast milk, and infant measures of vitamin B-12 status. J Nutr (2014) 144:758–64. 10.3945/jn.113.187278 PMC3985831 24598885

[19] Kessler RC Barker PR Colpe LJ Epstein JF Gfroerer JC Hiripi E Screening for serious mental illness in the general population. Arch Gen Psychiatry (2003) 60:184–9. 10.1001/archpsyc.60.2.184 12578436

[20] Fernandes MC Srinivasan K Stein AL Menezes G Sumithra RS Ramchandani PG Assessing prenatal depression in the rural developing world: a comparison of two screening measures. Arch Womens Ment Health (2011) 14:209–16. 10.1007/s00737-010-0190-2 21061137

[21] Malda M There is no place like home: on the relation between culture and children’s cognition. Ridderprint (2009).

[22] Rao K Subbakrishna DK Prabhu GG Development of a Coping Checklist—A Preminary Report. Indian J Psychiatry (1989) 31:128–33. PMC2991673 21927370

[23] Rao K Apte M Subbakrishna DK Coping and subjective wellbeing in women with multiple roles. Int J Soc Psychiatry (2003) 49:175–84. 10.1177/00207640030493003 14626360

[24] Bharathi AV Kurpad AV Thomas T Yusuf S Saraswathi G Vaz M Development of food frequency questionnaires and a nutrient database for the Prospective Urban and Rural Epidemiological (PURE) pilot study in South India: methodological issues. Asia Pac J Clin Nutr (2008) 17:178–85. 18364343

[25] Nasreen HE Kabir ZN Forsell Y Edhborg M Prevalence and associated factors of depressive and anxiety symptoms during pregnancy: a population based study in rural Bangladesh. BMC Womens Health (2011) 11:22. 10.1186/1472-6874-11-22 21635722 PMC3117808

[26] Yanikkerem E Ay S Mutlu S Goker A Antenatal depression: prevalence and risk factors in a hospital based Turkish sample. JPMA J Pak Med Assoc (2013) 63:472–7. 23905444

[27] Leigh B Milgrom J Risk factors for antenatal depression, postnatal depression and parenting stress. BMC Psychiatry (2008) 8:24. 10.1186/1471-244X-8-24 18412979 PMC2375874

[28] Srinivasan N Murthy S Singh AK Upadhyay V Mohan SK Joshi A Assessment of burden of depression during pregnancy among pregnant women residing in rural setting of Chennai. J Clin Diagn Res JCDR (2015) 9:LC08–12. 10.7860/JCDR/2015/12380.5850 PMC4437087 26023573

[29] Rahman A Iqbal Z Harrington R Life events, social support and depression in childbirth: perspectives from a rural community in the developing world. Psychol Med (2003) 33:1161–7. 10.1017/S0033291703008286 14580070

[30] Raman S Srinivasan K Kurpad A Dwarkanath P Ritchie J Worth H ‘My Mother…My Sisters… and My Friends’: sources of maternal support in the perinatal period in urban India. Midwifery (2014) 30:130–7. 10.1016/j.midw.2013.03.003 23561829

[31] O’Leary N Jairaj C Molloy EJ McAuliffe FM Nixon E O’Keane V Antenatal depression and the impact on infant cognitive, language and motor development at six and twelve months postpartum. Early Hum Dev (2019) 134:41–6. 10.1016/j.earlhumdev.2019.05.021 31176100

[32] Murray L Kempton C Woolgar M Hooper R Depressed mothers’ speech to their infants and its relation to infant gender and cognitive development. J Child Psychol Psychiatry (1993) 34:1083–101. 10.1111/j.1469-7610.1993.tb01775.x 8245134

[33] Paulson JF Keefe HA Leiferman JA Early parental depression and child language development. J Child Psychol Psychiatry (2009) 50:254–62. 10.1111/j.1469-7610.2008.01973.x 19175819

[34] Stein A Lehtonen A Harvey AG Nicol-Harper R Craske M The influence of postnatal psychiatric disorder on child development. Is maternal preoccupation one of the key underlying processes? Psychopathology (2009) 42:11–21. 10.1159/000173699 19023230 PMC2705013

[35] Kostović I Jovanov-Milosević N The development of cerebral connections during the first 20-45 weeks’ gestation. Semin Fetal Neonatal Med (2006) 11:415–22. 10.1016/j.siny.2006.07.001 16962836

[36] Andescavage NN du Plessis A McCarter R Serag A Evangelou I Vezina G Complex trajectories of brain development in the healthy human fetus. Cereb Cortex (2017) 27:5274–83. 10.1093/cercor/bhw306 PMC6074870 27799276

[37] Srinivasan K Thomas S Anand S Jayachandra M Thomas T Strand TA Vitamin B-12 supplementation during pregnancy and early lactation does not affect neurophysiologic outcomes in children aged 6 years. J Nutr 150(7):1951–7. 10.1093/jn/nxaa123 PMC7330478 32470975

[38] Sparling TM Nesbitt RC Henschke N Gabrysch S Nutrients and perinatal depression: a systematic review. J Nutr Sci (2017) 6:e61. 10.1017/jns.2017.58 29296279 PMC5738654

[39] Trujillo J Vieira MC Lepsch J Rebelo F Poston L Pasupathy D A systematic review of the associations between maternal nutritional biomarkers and depression and/or anxiety during pregnancy and postpartum. J Affect Disord (2018) 232:185–203. 10.1016/j.jad.2018.02.004 29494902

[40] Ranjitkar S Kvestad I Strand TA Ulak M Shrestha M Chandyo RK Acceptability and reliability of the bayley scales of infant and toddler development-III among children in Bhaktapur, Nepal. Front Psychol (2018) 9:1265. 10.3389/fpsyg.2018.01265 30087639 PMC6066572

[41] McDonald CM Manji KP Kupka R Bellinger DC Spiegelman D Kisenge R Stunting and wasting are associated with poorer psychomotor and mental development in HIV-exposed Tanzanian infants. J Nutr (2013) 143:204–14. 10.3945/jn.112.168682 PMC3542911 23256148

